# The implication of dendritic cells in lung diseases: Immunological role of toll-like receptor 4

**DOI:** 10.1016/j.gendis.2023.04.036

**Published:** 2023-06-27

**Authors:** Shurui Xuan, Yuan Ma, Honglei Zhou, Shengwei Gu, Xin Yao, Xiaoning Zeng

**Affiliations:** a Department of Pulmonary & Critical Care Medicine, The First Affiliated Hospital of Nanjing Medical University, Nanjing, Jiangsu 210029, China; b Department of Respiratory Medicine, Nanjing First Hospital, Nanjing Medical University, Nanjing, Jiangsu 210006, China; c Department of General Surgery, The First Affiliated Hospital of Nanjing Medical University, Nanjing, Jiangsu 210029, China

**Keywords:** Dendritic cells, Immunity, Lung diseases, Pathogen recognition receptors, Toll-like receptors 4

## Abstract

The immune responses play a profound role in the progression of lung lesions in both infectious and non-infectious diseases. Dendritic cells, as the “frontline” immune cells responsible for antigen presentation, set up a bridge between innate and adaptive immunity in the course of these diseases. Among the receptors equipped in dendritic cells, Toll-like receptors are a group of specialized receptors as one type of pattern recognition receptors, capable of sensing environmental signals including invading pathogens and self-antigens. Toll-like receptor 4, a pivotal member of the Toll-like receptor family, was formerly recognized as a receptor sensitive to the outer membrane component lipopolysaccharide derived from Gram-negative bacteria, triggering the subsequent response. Moreover, its other essential roles in immune responses have drawn significant attention in the past decade. A better understanding of the implication of Toll-like receptor 4 in dendritic cells could contribute to the management of pulmonary diseases including pneumonia, pulmonary tuberculosis, asthma, acute lung injury, and lung cancer.

## Introduction

As a human organ in extensive contact with external substances, the lung has a strong innate immune system for the recognition and defense against a wide array of pathogenic and environmental substances, such as bacteria, viruses, and air pollution.^1^ Dendritic cells (DCs) are vital cell components of innate immunity, playing crucial roles in the development of adaptive immune responses.^2^ Within the lung, DCs are located in the mucosa of bronchioles and alveoli, as well as lymph nodes around the airway,^3^, ^4^, ^5^ which are equipped with pattern recognition receptors (PRRs) recognizing and responding to specific patterns of molecules associated with pathogens or damaged cells. There are several different types of PRRs, including Toll-like receptors (TLRs), NOD-like receptors (NLRs), RIG-I-like receptors (RLRs), and C-type lectin receptors (CLRs). As a member of the TLR family of PRRs, TLR4 has been identified as the receptor responsive to lipopolysaccharide (LPS), which is a component of the outer membrane of Gram-negative bacteria. After the binding of TLR4 and LPS on the surface, DCs migrate from the mucosa to the nearest lymph nodes, up-regulating the costimulatory molecules and cytokine secretion. Moreover, the lung is highly vascularized, which facilitates the recruitment of DCs or differentiation of precursor cells from the bloodstream triggering the elimination of invading pathogens in the lung. The recruitment and differentiation process contribute to the immune defense and homeostasis in the lung.^6^ Upon activation, DCs “talk” with naïve CD4^+^ T cells, CD8^+^ T cells, and B cells to present antigenic information, triggering the initiation of adaptive immunity.^7^ In addition to LPS, other ligands for TLR4 have been identified, such as cystatin^8^ and heat-shock protein.^9^ However, the exact role of TLR4 in DCs involved in immune responses remains obscure. Herein, we focus on the studies of TLR4 in DCs in immune-related lung diseases. Their critical roles in various lung diseases including pneumonia, pulmonary tuberculosis (TB), asthma, acute lung injury (ALI), and lung cancer, have been identified and highlighted, providing important clues for the development of new clinical strategies.

## The expression and characteristics of TLR4 in DCs in the lung

Generally, DCs can be classified into several subsets in the lung, including plasmacytoid DCs (pDCs), monocyte-derived DCs (moDCs), and classical (or conventional) DCs (cDCs), which can further be divided into type 1 and type 2 subtypes.^10^ As indicated in Table 1, each subset expresses different TLRs with distinct surface markers.^10^ There are a total of 15 members of TLRs identified in mammals, and 10 of them (TLR1–10) are present in humans. Through nucleic acid sensors (TLR3 and TLR9) and lipid sensors (TLR2 and TLR4), cDCs contribute to the cross-presentation of diverse antigens.^7^ Monocytes serve as the primary source for DC replenishment and differentiate into moDCs by expressing most types of TLRs (except TLR9 and TLR10) upon sensing stimulus signals.^11^ Additionally, pDCs play a crucial role in antiviral immunity and express nucleic acid-sensing receptors TLR7 and TLR9. They produce substantial amounts of type I IFN (IFN-α and/or IFN-β) in response to TLR7/9 signals and are thus implicated in autoimmune diseases.^12^

**Table 1:** TLRs of specific DC subsets in the human airway.

DC subset	Surface markers	TLRs	Immunological processes/functional specialization	Reference(s)
Lung cDC1	CD1a^−^CD1c^+^	TLR2/3/4/9	Airway tolerance to harmless inhaled antigens; Th1 and Th2 polarization; Th17 response during sensitization against *Aspergillus fumigatus*	13, 14, 15, 16, 17
	CD11b^+^CD11c^+^			
	CD14^−^CD123^−^			
Lung cDC2	CD1a^−^CD11b^−^	TLR2/3/9	Airway tolerance to harmless inhaled antigens; Th1 and Th2 polarization	18
	CD11c^+^CD14^−^			
	CD123^−^CD141^+^			
Lung moDC	CD1a^+^CD1c^+^	TLR1/2/3/4/5/8	Proinflammatory cytokine production and allergen presentation during secondary antigen stimulation; attraction of monocytes to the infected side; restimulation of memory Th1 cells after secondary antigen stimulation	18, 19, 20
	CD11b^+^CD11c^+^			
	CD14^−^CD64^+^			
	CD123^−^CD141^−^			
Lung pDC	CD3^−^CD4^+^	TLR7/9	Airway tolerance to harmless inhaled antigens; produce a large amount of IFN-α upon activation; induction of Treg cells	21, 22, 23, 24, 25
	CD11b^−^CD11c^−^			
	CD31^+^CD45RA^+^			
	CD68^+^CD123^+^			

As a crucial receptor involved in innate immunity, TLR4 has been identified in cDCs, but not in pDCs.^7^ It performs a functional role in CD8^+^ cDCs compared to CD141^+^ cDCs.^26^^,^^27^ TLR4 acts as a vital sensor of LPS and it is activated with signals further amplified by a series of events. The activation process begins from the extracellular side of DCs where LPS monomers are obtained from aggregates in solution and transferred to LPS-binding protein (LBP). Subsequently, the monomers are delivered to the cluster of differentiation 14 (CD14), which is either GPI-linked or soluble, and then to myeloid differentiation factor 2 (MD2), also referred to as LY96, which noncovalently associates with TLR4.^28^ These sequential events initiate a signaling cascade that potentiates the function of DCs.

**Figure 1:**
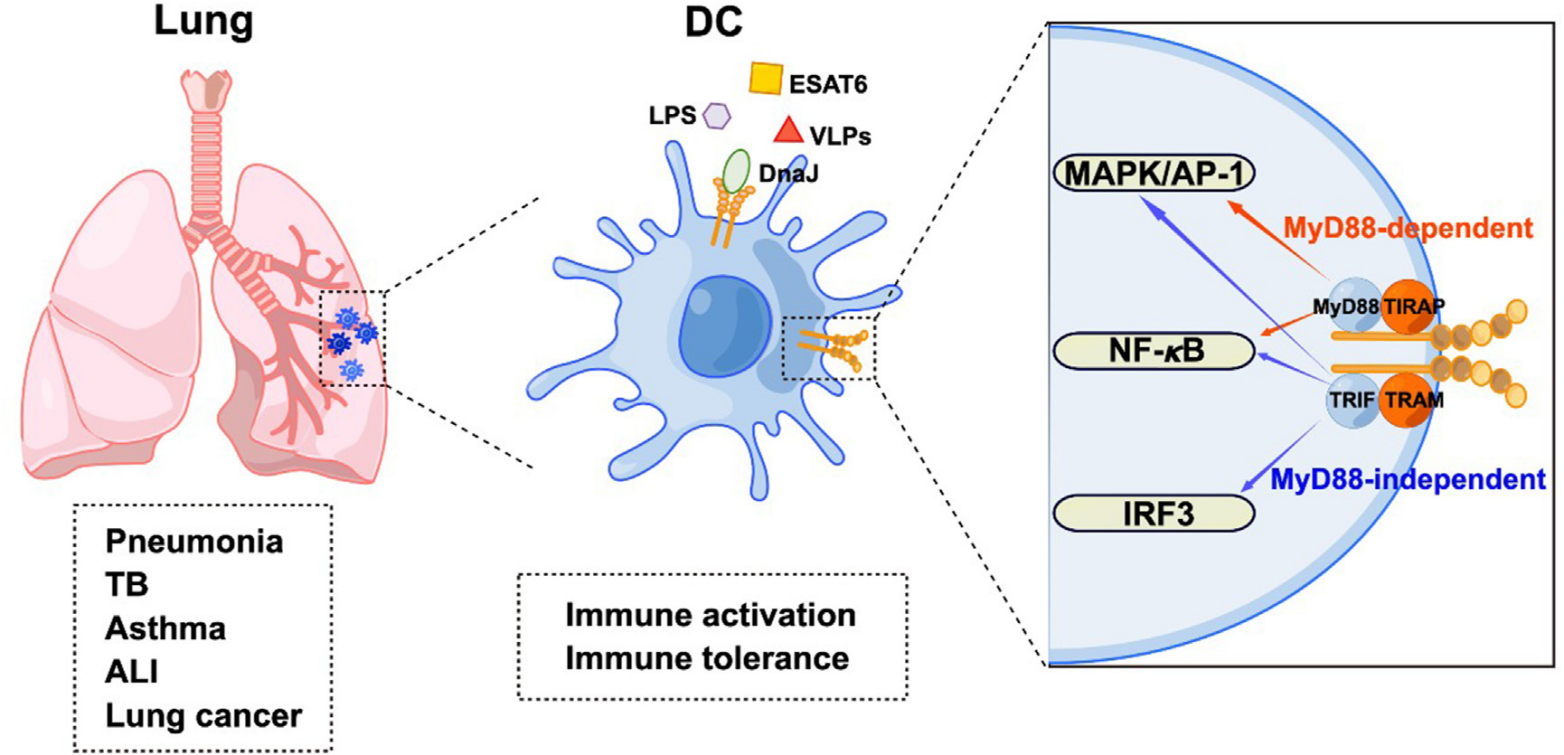
Involvement of TLR4 signal in dendritic cells (DCs) in inflammatory lung diseases.

Signaling of TLR4 in DCs has been classified into myeloid differentiation factor 88 (MyD88)-dependent and MyD88-independent way. MyD88-dependent and -independent pathways exhibit differences in TLR4-related infectious and non-infectious diseases (Table 2). In infectious diseases, the primary pathway utilized by the host to respond to the pathogen and clear the infection is typically MyD88-dependent TLR4 signaling, which is required for the host to defend against Gram-negative bacterial infections such as *Klebsiella pneumoniae* (*K. pneumoniae*)^29^ and *Pseudomonas aeruginosa* (*P. aeruginosa*).^30^ Conversely, in non-infectious diseases, TLR4 signaling activation occurs via endogenous ligands like damage-associated molecular patterns (DAMPs) and danger signals, released during tissue damage, stress, or chronic inflammation.^31^ In such situations, MyD88-independent TLR4 signaling primarily triggers the inflammatory response. MyD88-dependent mechanism carries out the activation of nuclear factor kappa-B (NF-κB) and activator protein (AP)-1, while MyD88-independent signaling involves TIR-domain-containing adaptor inducing interferon B-dependent (TRIF) and triggers interferon regulatory factor 3 (IRF3) activation.^28^ Additionally, the TLR4 signaling response can be affected by various factors, including genetic variations, environmental factors, microbial diversity, age, and health status (Table 2). The complication of this enormous regulatory network hints at the involvement of TLR4 in both exogenous and endogenous inflammation, participating in infectious and non-infectious lung diseases including pneumonia, pulmonary TB, asthma, ALI, and lung cancer (Fig. 1). Since the clinical application and efficacy of immunotherapies for these diseases present quite limited by far, improved understanding of the role of TLR4 in airway DC activation is helpful for the innovation of promising strategy for these pulmonary diseases.

**Table 2:** The differences between the MyD88-dependent and -independent pathways and driven factors of TLR4 signaling.

Pathways	Driven factors	Lung diseases	Reference(s)
MyD88-dependent pathway	Environmental factors, *e.g.*, microbial products	Pneumonia, tuberculosis, asthma	32, 33, 34
	Microbial diversity	Acute lung injury	35
MyD88-independent pathway	Genetic variations, *e.g.*, SNPs	Chronic obstructive pulmonary disease	36
	Environmental factors, *e.g.*, pollutants, microbial products, and dietary factors	Asthma	37
	Age, health status	Pulmonary fibrosis	38

## DC TLR4 in infectious inflammatory lung diseases

### Gram-positive bacteria

Bacterial pneumonia is a prevalent category of pulmonary infectious diseases. Based on Gram staining, bacterial pathogens are typically classified as either Gram-positive or Gram-negative bacteria. Gram-positive bacteria, including *Streptococcus pneumoniae* (*S. pneumoniae*) and *Staphylococcus aureus* (*S. aureus*), are the most common causes of community-acquired and nosocomial pneumonia. Although the components of Gram-positive bacteria, lipoteichoic acid,^39^ and peptidoglycan^40^ were reported to agonize TLR2 commonly, TLR4 in DCs also plays a crucial role in *S. pneumoniae* infection.

As a highly conserved cell surface protein found in various serotypes of pneumococcal strains, DnaJ has been identified as a potential protein vaccine antigen. DnaJ can activate and mature bone marrow-derived dendritic cells (BMDCs) in a TLR4-dependent manner.^41^ The maturation involves mitogen-activated protein kinases (MAPKs), phosphatidylinositol 3-kinase (PI3K)-Akt and NF-κB pathways, and then induce naïve CD4^+^ T cell differentiation toward Th1 and Th17 polarizing response which is TLR4-dependent as well.^41^ These findings suggest that TLR4 is protective against *S. pneumoniae* infections through the intrinsic immunogenicity of DnaJ protein. Similarly, another research team identified the housekeeping protein glyceraldehyde-3-phosphate dehydrogenase (GAPDH) on the surface of *S. pneumoniae*, which also activates BMDCs through a TLR2/TLR4 mechanism.^42^ In a mouse model of post-hemorrhage pneumonia induced by methicillin-susceptible *S. aureus*, Roquilly et al showed that monophosphoryl lipid A (a TLR4 agonist inducing interferon-biased response) partially recovered antigen presentation and transcriptional activity in DCs and reduced systemic *S. aureus* spread and attenuated inflammatory lung lesions after hemorrhage damage.^43^ In summary, TLR4 mediates positive immune promotion against Gram-positive infections, including DC activation and subsequent inflammatory response.

### Gram-negative bacteria

Compared to Gram-positive bacterial pathogens, TLR4 takes more engagement in host immunity against Gram-negative bacteria. Epidemiologically common Gram-negative bacteria include *K. pneumoniae*, *P. aeruginosa*, and *A. baumannii*. A major component of their outer membrane, endotoxin, and its purified derivative LPS are potent TLR4 agonists continuously shed into the surrounding environment.^44^^,^^45^ The cooperative interaction between the LPS-binding protein, CD14, and the TLR4-MD2 complex initiates LPS recognition of TLR4 in innate immune cells.^46^ Upon activation by LPS, TLR4 involves in a signaling pathway that includes interleukin (IL)-1, tumor necrosis factor receptor-associated factor (TRAF)-6, and NF-κB.^47^ Flow cytometry analysis of induced sputum cells indicates that acute LPS inhalation enhances DC maturation in healthy volunteers directly through TLR4 stimulation or indirectly through cytokine stimulation.^48^ Compared to Gram-positive bacteria, Gram-negative bacteria are capable of inducing podosome (specialized adhesion structures found in immature DCs) dissolution which is dependent on TLR4 and downstream signals and is necessary for the induction of effective DC migration.^49^ Additionally, Gram-negative bacteria can induce the production of immunogenetic cytokines. *P. aeruginosa* and LPS activate murine BMDCs to induce type I IFN gene products via a TLR4/TRIF/MD2 cascade. Kim and coworkers showed that TLR4-deficient BMDCs partially reduced the production of IL-6, TNF-α, and IL-12p40 in response to *A. baumannii*.^50^

Mature DCs migrate from tissue to lymph nodes, where they deliver antigens to naïve T lymphocytes. Pathogen components, such as *Escherichia coli* (*E. coli*) LPS, activate TLR4 and induce IL-12 production by innate immunity cells, *e.g.*, DCs, directing the activation of Th1 cells.^51^ A further study has reported that *E. coli* LPS triggered TLR4 and instructed DCs to produce IL-12p70 through phosphorylating p38 and c-Jun N-terminal kinase (JNK) 1/2, thereby stimulating Th1 responses.^52^ The lysate of the outer membrane fraction of *K. pneumoniae*, containing LPS and outer membrane protein A (OmpA), triggers DCs respectively through TLR4 and TLR2, then leading to the recruitment of natural killer (NK) cells in a CCR5-dependent manner.^53^ The enhanced cytotoxicity of NK cells and IFN-γ secretion may contribute to Th1 polarization.^53^ Different antigens expressed by the same bacterium can also have varying effects on DCs. In contrast to DC activation by LPS O antigen, the capsular polysaccharide (CPS) of *K. pneumoniae* induced a defective immunological host response by hindering bacterial binding and internalization.^54^ In addition to cellular immunity, DCs can activate humoral immunity. In a mouse lung co-culture model of DCs and B cells, *Mycoplasma hyopneumoniae* triggered the response of immunoglobulin A (IgA) via TLR2/TLR4.^55^ Although the specific mechanism was not studied, the authors speculate that the capsular component of *Mycoplasma pneumoniae* can promote the secretion of IL-10 by activating TLR4 in DCs, thereby triggering IgA production in B cells.

TLR4 plays a vital role not only in promoting protective cellular immunity but also in facilitating immunosuppression to prevent collateral damage caused by pathogen-induced inflammatory responses. The recovery of *Bordetella pertussis* infection has revealed that DCs produce IL-10 through TLR4, leading to the activation of Treg cells and serving as a negative immune regulation mechanism that is protective for the host.^56^ TLR4-mediated DC flexibility in generating either inflammation or tolerance can be affected differently *in vitro* by single versus repeated exposure to LPS. Following repeated stimulation with LPS, cDCs exhibit higher levels of the anti-inflammatory mediator indoleamine 2,3-dioxygenase 1 (IDO1) and the regulatory cytokine transforming growth factor (TGF)-β. However, single LPS exposure up-regulates IL-6, promoting inflammation and proteolysis of IDO1.^57^ These findings suggest that low doses of LPS may induce DC-mediated endotoxin tolerance and that the transfer of tolerance may represent a novel strategy for regulating TLR4-mediated inflammatory responses.

Of interest, the regulation of TLR4-mediated responses of DCs involves various factors that influence immune enhancement or suppression. For instance, the type of ligand that binds to TLR4, the intensity and duration of the TLR4 signaling,^58^ and the presence of co-receptors or co-stimulatory molecules^32^ may all impact the immune response. Furthermore, environmental factors such as diet,^59^^,^^60^ microbiota, and pollutants have also been found to influence TLR4-mediated immune responses by mediating TLR4 ligand recognition and signal transduction. Numerous studies have shown that a series of regulatory factors are involved in the TLR4-mediated LPS-induced pulmonary inflammation of DCs. Within the immune regulatory network, various receptors interact extensively with one another. Surfactant protein-A (SP-A) is a secretory pathogen recognition receptor produced by epithelial cells on the mucosal surface.^61^ Awasthi et al reported that TLR4-interacting SP-A peptides interact with TLR4-MD2 protein and inhibit the LPS-induced release of TNF-α in the mouse JAWS II DC line.^62^ Furthermore, the authors have supported and expanded their findings on the region from the interface of SP-A-TLR4 complex identified as SPA4 peptide through multiple *in vitro* and *in vivo* experiments.^63^ Additionally, in a mouse model of *P. aeruginosa* lung infection, the researchers have demonstrated that the therapeutic administration of SPA4 peptide induces the uptake and localization of bacteria in immune cells, including JAWS II DCs, which subsequently reduces the bacterial burden, suppresses inflammatory cytokines/chemokines and lactate levels, inhibits intracellular signal transduction, thus alleviates lung edema and tissue damage.^64^

The activation of TLR4 in DCs by LPS is preceded by a series of reactions initiated by serum LBP, which converts LPS aggregates into highly concentrated LPS monomers at the cell surface. These monomers were subsequently extracted by CD14 and then delivered to the TLR4/MD2 complex.^65^, ^66^, ^67^ CD14 is anchored in cholesterol and sphingolipid-rich plasma membrane microdomains known as rafts.^68^ It has been discovered that a decrease in CD14 cell surface levels is the primary cause of defective TLR4 endocytosis.^69^ Conversely, the up-regulation of CD14 during the maturation of murine DC accelerates LPS-induced TLR4 endocytosis.^70^ In addition to controlling the macropinocytosis of LPS-activated TLR4 through CD14 in DCs, CD14 has an additional role. The raft localization of CD14 is essential for its function in LPS-stimulated DCs, as indicated by CD14-dependent activation of nuclear factor of activated T cells (NFAT). Ca2^+^ influx induced by LPS stimulation of CD14 leads to calcineurin-mediated activation of NFAT in DCs. Subsequently, IL-2 and prostaglandin E2 production surge, and DCs undergo apoptosis.^71^^,^^72^ Along with CD14, other raft proteins such as Lyn tyrosine kinase of the Src family^73^^,^^74^ participate in LPS-triggered TLR4 signaling in DCs as well.

In addition to serving as sensors of exogenous or foreign PAMPs, TLR4 in DCs may also identify and modulate responses to endogenous stimuli, such as the heat shock protein 60 (HSP60) secreted by necrotic cells undergoing cell death^75^ and fibrin generated from plasma-derived fibrinogen.^76^ Thus, severe tissue injury may stimulate endogenous TLR4 activation, leading to an enhanced TLR4-mediated proinflammatory response by DCs.^77^ TLR4 agonists hold promise as effective adjuvants for *Brucella abortus* (*B. abortus*) vaccines. The *B. abortus* strain RB51 has been reported to promote DC activation, increase IgG and IgA in the bronchoalveolar lavage (BAL) or serum, and enhance CD4^+^ Th1 and CD8^+^ Tc1 immune responses.^78^

### Mycobacterium tuberculosis

Tuberculosis (TB), caused by acid-fast stain-positive *Mycobacterium tuberculosis* (*Mtb*), is a major source of illness and a leading infectious cause of mortality worldwide, ranking above HIV/AIDS.^79^ During *Mtb* infection, DCs play a significant role in the development and direction of adaptive immunity by presenting mycobacterial antigens and expressing costimulatory signals and cytokines.^80^ A series of studies have been conducted to investigate the pathophysiological mechanism of DC activation by *Mtb*. As a virulent factor of *Mtb*,^81^^,^^82^ the 6-kDa early secretory antigenic target (ESAT6) drives the activation and maturation of BMDCs via TLR4-mediated signaling.^83^ The maturation of DC is characterized by the up-regulation of costimulatory molecules and enhanced cytokine release. Additionally, in a study of human peripheral blood and a mouse model, *Mtb* components stimulate the production of hepcidin from DCs, which is an antimicrobial peptide with broad-spectrum antimicrobial activities.^84^ The mycobacterial cell wall contains glycolipid macromolecules that have significant and distinct effects on human DCs. For example, mannose-capped lipoarabinomannan (ManLAM) is immunostimulatory, while phosphatidylinositol mannosides (PIMs) function as potent inhibitors of DC cytokine responses.^85^

Furthermore, additional studies on the adaptive immunity induced by DC activation have shown that *Mtb*GrpE (a heat-shock stress-responsive chaperone),^86^ *Mtb* protein resuscitation-promoting factor B (RpfB, a secretory protein),^87^ *Mtb* protein Rv3841 (bacterioferritin B, BfrB),^88^ and PPE39 (a protein in *Mtb* strain Beijing/K)^89^ stimulate Th1-type T cell immunity through TLR4-dependent activation of DCs. In addition to the Th1-biased T cell immune response, RpfE contributes to a Th17 immune response via TLR4 binding and subsequent activation of MAPK (ERK and p38) and NF-κB signaling.^90^ In tuberculous pleural effusions and peripheral blood from TB patients compared to healthy controls, an increased CD1c DC subset with the phenotypic CD1c^+^ CD11c^+^ CD19^−^ CD11b^+^ and a significantly enhanced TLR4 expression level was identified.^91^ When co-cultured with autologous naïve CD4^+^ T cells, the CD1c^+^ CD11b^+^ DC subset stimulated the generation of Th17 cells.^91^ Moreover, TLR4 can enhance the activation of T cells by mediating DC autophagy and inducing DC maturation. Agrewala et al observed that NOD-like receptor-2 (NOD-2) and TLR-4 act in concert to trigger autophagy in DCs, thereby enhancing the DC capacity to activate T cells.^92^ They applied this finding in a mouse model of TB and demonstrated that it not only boosted the immune system but also decreased the dosage and duration of rifampicin and isoniazid treatment.^93^ These findings suggest that NOD-2 and TLR-4 signals in DCs are potential therapeutic targets that can reinforce host immunity and improve TB treatment outcomes.

The response against *Mtb* of DCs is regulated by diverse *in vitro* and *in vivo* factors. Corresponding experiments indicated that the MyD88-mediated signaling pathway is a crucial element for the establishment of innate immune responses, exemplified by TNF-α and IL-12 p40 production from DCs against *Mtb* infection. However, adaptive immunity and TH1 responses are still observed even in the absence of MyD88.^94^ The lack of IL-1R signaling demonstrates a similar impairment of early *Mtb* infection control, suggesting that IL-1 and IL-1-induced innate immunity play a substantial role in the MyD88-dependent host response during acute *Mtb* infection.^95^ Immunomodulatory factors, such as six-O-acyl-muramyldipeptides (monoacylated MDP), BCG-cell wall skeleton (CWS), and BCG-peptidoglycan (PGN), can serve as beneficial adjuvants for the Calmette-Guerin (BCG) vaccine in clinical settings. The activation patterns of human DCs by six-O-acyl MDP were comparable to that by CWS and PGN which was demonstrated by their ability to up-regulate costimulators, HLA-DR, β2-microglobulin, and allostimulatory monocyte/lymphocyte ratio (MLR). Six-O-acyl MDP is a potential adjuvant that targets TLR2/4 pathways through MyD88-dependent signaling pathways in DCs to induce cytokine profiles including TNF-α, IL-12p40, and IL-6.^96^ Glucopyranosyl lipid adjuvant-stable emulsion (GLA-SE), a synthetic TLR4 agonist type adjuvant has also shown its efficacy. Paired with the TB vaccine antigen ID93, GLA-SE promotes a Th1 immune response characterized by CD4^+^ T cells generating IFN-γ, TNF-α, and IL-2, as well as IgG2c class switching through the MyD88 and TRIF synergistic interaction.^97^ Lin et al discovered that the macrolide antibiotic azithromycin (AZM) can down-regulate CD80, CD86, and HLA-DR expression and suppress the production of IL-6, IL-10, IL-12, and TNF-α in LPS-stimulated DCs. Moreover, AZM can enhance endocytosis and/or expression of TLR4 in DCs and suppress the proliferation of CD4^+^ T cells and the production of IFN-γ, indicating its potential therapeutic use.^98^ Additionally, *E. coli* bacterial ghosts (BGs) which are complete Gram-negative bacterial cell envelopes without cytoplasm generated by the conditional expression of plasmid-encoded gene E from the bacteriophage ΦX174, have been shown to have therapeutic adjuvant properties against TB.^99^ BG can activate markers, partially dependent on TLR4, increase the migratory ability of DCs, and promote T cell differentiation into CD4^+^ Th1 lineage. *In vivo*, treatment with BG decreases lung bacterial burden in mice infected with *M. bovis* BCG and *Mtb* and synergizes with second-line agents bedaquiline (BDQ) and delamanid (DLM).^100^

### Virus pathogens

In addition to bacteria, various viral pathogens, such as Ebola virus (EBOV), human metapneumovirus (hMPV), influenza virus, and respiratory syncytial virus (RSV), can modulate immune responses through TLR4.

*In vivo*, DCs are important early targets of EBOV infection and are activated by Ebola virus-like particles (VLPs) containing the viral matrix protein (VP40) and viral glycoprotein (GP), leading to the production of a number of proinflammatory cytokines and the activation of several transcription factors, including NF-κB and ERK1/2 MAPK.^101^ Further research on a human monocytic cell line (THP-1 cells) reveals that Ebola virus VP40^+^ GP VLPs induce cytokines (TNF-α, IL-6, and IFN-β) and suppressor of cytokine signaling 1 (SOCS1) expression in a TLR4/MD2 dependent manner.^102^ Shed GP and non-structural secreted glycoprotein sGP are both present in the blood of infected humans and animals. Shed GP functions by binding and activating non-infected DCs, leading to the release of pro- and anti-inflammatory cytokines (TNF-α, IL-1β, IL-6, IL-8, IL-12p40, IL-1-RA, and IL-10) and an increased expression of costimulatory molecules CD40, CD80, CD83, and CD86 on the surface of DCs, whereas sGP can bind to DCs without activating them.^103^ In mice lacking TLR4 and infected with hMPV, decreased levels of proinflammatory cytokines (IL-1β, IL-6, and TNF-α), immunomodulatory cytokines (GM-CSF, IL-12 p40, IL-17), and chemokines (MCP-1, MIP-1α) were observed, along with impaired DC-mediated T cell proliferation. It indicated the essential role of TLR4 in the activation of innate rather than adaptive immunity against hMPV infection.^104^ Casola et al have previously demonstrated that TLR4 takes an essential part in hMPV G glycoprotein-induced activation of mDCs, inducing chemokine and type I IFN expression.^105^ Influenza virus infection induces the expression of cytokines (*i.e.*, TNF-α, IL-1β, and IFN-β) and chemokines such as KC (murine IL-8). While TLR4 antagonists Eritoran^106^ and FP7^107^ are highly protective when delivered therapeutically to mice infected with a fatal dose of influenza virus (A/PR/8/34, known as PR8). Young infants are particularly susceptible to RSV infections, and a neonatal murine model of RSV infection has shown that treatment with TLR4 agonists significantly promotes an adult-like CD8^+^ T cell response, as well as increasing the quantity of cDCs and the expression of the costimulatory molecule CD86 in the lung draining lymph nodes.^108^ Additionally, the involvement of TLR4 agonists has been shown to enhance immune responses as potent adjuvants when administered with subunit vaccines.^109^ Th17 cells are absent in healthy neonates but present in infants with acute RSV infection, and TLR4-mediated Th17-polarizing DC response is responsible for their promotion.^110^ Overall, TLR4 expressed in DCs plays a crucial role in promoting the activation of both innate and adaptive immunity.

## DC TLR4 in non-infectious inflammatory lung diseases

### Asthma

Current researches present a contentious debate regarding the role of TLR4 signaling in regulating allergic inflammation. Notably, asthmatic patients were found to exhibit a reduced expression level of TLR4 in DC population in comparison to control subjects. Consequently, the authors hypothesized that the TLR4-related Th1 profile may be diminished.^111^ Similarly, a lower expression of TLR4 was observed in the mDCs of relapsed eosinophilic granulomatosis with polyangiitis (EGPA) patients relative to EGPA patients in remission or non-EGPA patients. While TLR4 was positively associated with the percentages of Treg and Th17 cells.^112^

On the other hand, numerous studies have put forth the argument that TLR4 in DCs plays a crucial role in promoting immunity. In mice, protease allergens or fibrinogen cleavage products (FCPs) were found to cause a significant increase in Th2-favorable and TLR4-dependent programmed cell death 1 ligand 2 (PD-L2)^+^ DCs in mediastinal lymph nodes.^113^ Inhalation of house dust mite (HDM) was observed to induce the TLR4/MyD88-dependent migration of IL-4 capable basophils and eosinophils, as well as inflammatory DCs, to the draining mediastinal lymph nodes.^34^ Allergen-specific IgG can enhance Th2-mediated responses by ligating FcγRIII (a receptor of IgG-immune complexes) and TLR4 in DCs through an IL-33-dependent pathway in lung.^114^ *In vitro*, high mobility group box 1 protein (HMGB1) may also enhance Th2 or Th17 differentiation by activating TLR2, TLR4, and RAGE-NF-κB signaling in DCs to regulate their maturation and antigen-presenting capacity.^115^

The role of TLR4 in DCs is critical in HDM-mediated allergic airway inflammation. HDM-derived extracellular vesicles were isolated, containing abundant amounts of LPS which may be immunogenic in the development of airway inflammation.^116^ The LPS-binding protein MD2, implicated in LPS-induced TLR4 signaling, protects against HDM-induced airway allergy by regulating inflammation in airway epithelial cells and subsequently activating DCs.^117^ The main HDM allergen, Derp2, has structural similarity with MD2 in the TLR4 signaling complex.^118^, ^119^, ^120^ Immunoaffinity-purified Derp2 from HDM induced TLR4-dependent TNF-α secretion by mouse BMDCs.^121^ In MD2-null mice, Derp2 could also activate DCs,^121^ mimicking the TLR4-activating properties of LPS-MD2 complexes.^122^ The divergent abundance of LPS in HDM extracts was reported to induce significantly different cytokine profiles of bronchial lavage and differential gene enrichment based on whole lung transcriptome analysis.^123^ It was demonstrated that LPS dose-dependently inhibited HDM-induced eosinophil recruitment, attenuated the production of Th2 cytokines (IL-4, IL-5, IL-10, and IL-13), and enhanced the release of IL-6, IL-17, IL-33, IFN-γ, and TNF-α, particularly at a relatively higher dose (10 μg).^124^ Bachus et al challenged HDM-sensitized mice with relatively higher (50 μg) and lower doses (5 μg) of LPS. The lower dose prevented allergic Th2 cell responses in lymph nodes as CD11b^+^ mDCs up-regulated the transcription factor T-bet in responding T cells after direct recognition of LPS by TLR4.^125^ The research team further unraveled the underlying mechanism of cytokine regulation in the dual effect of LPS, either inducing or suppressing Th2 cell responses. In the presence of GM-CSF, moDCs with programming enhanced TLR4 expression promoted lung mDC2s producing IL-12 to prevent Th2 cell priming. In the absence of GM-CSF and the lack of moDCs, LPS exposure promoted Th2 cell-mediated immunity.^126^

Besides the HDM sensitization model, the OVA model is a well-established method of inducing asthma. In mouse models, airway sensitization with antigen OVA, combined with a very low-dose (<1 ng) LPS, induces tolerance. Conversely, low-dose (100 ng) LPS triggers Th2 immune responses and allergic asthma in a TLR4-dependent manner.^127^ It is interesting to note that in neonatal asthmatic mice, pre-exposure to LPS (1 μg) induces tolerogenic Treg skewing by down-regulating TLR4-dependent TRIF/IRF3/IFNβ-mediated glucocorticoid-induced tumor necrosis factor receptor ligand (GITRL, a Treg-suppressive factor) in DCs.^128^

In contrast, airway sensitization with OVA plus high-dose (100 μg) LPS induces a Th1 response, which suppresses the generation and activation of Th2 immunity.^127^^,^^129^ Studies have shown that in addition to inducing a Th1 response, LPS increases the selective enrichment of CD11b^+^ Gr1 (int)F4/80^+^ cells (referred to as Gr1 (int) cells) over DCs in the lung tissue of mice dependent on MyD88-mediated TLR4 signal, contributing to the immunosuppressive effect. Gr1 (int) cells were found to blunt the ability of lung DCs to up-regulate transcription factors involved in Th2 effector function, such as GATA-3 or STAT5 activation in primed Th2 cells.^130^ Coadministration of OprI (lipoprotein I from *P. aeruginosa*) and OVA intranasally led to a significant reduction in Th2 cytokines (IL-4 and IL-13) and an increase in Th1 cytokine IFN-γ production. OprI stimulated DCs to produce IL-12 and TNF-α, which subsequently triggered the production of IFN-γ from T cells via TLR2/4 signaling pathway.^131^ Dermal exposure to TLR4 ligand LPS or TLR2 ligand Pam3Cys suppressed asthmatic responses by reducing airway hyperreactivity, mucus production, Th2-type inflammation in the lung, and IgE antibodies in the serum of OVA-induced mouse model. LPS specifically augmented the activation of dermal DCs by increasing the expression of CD80 and CD86, diverting the Th1/Th2 balance toward Th1 by stimulating the production of IFN-γ.^132^ Marsland et al reported that exposure to the bacteria *E. coli* significantly suppresses Th2 responses characterized by reduced airway hyperreactivity, decreased eosinophilia, and cytokine production by T cells in the lung. The process consists of two pathways, inhibition of airway hyperreactivity by recruited γδ T cells and ineffective antigen presentation to TLR4-dependent effector T cells in the lung achieved by inhibiting DC activity.^133^ Co-absorption of TLR4 agonists (including LPS) onto alum impaired OVA-induced Th2-mediated allergic responses by activating TLR4-MyD88 signaling and the IL-12/IFN-γ axis.^134^ On the contrary, Rodríguez et al proved that LPS suppresses Th2 responses through TLR4 signaling via nitric oxide generated by nitric oxide synthases 2 independently of IL-12 or IFN-γ production.^135^ Although these studies did not construct conditional knockout mouse models, they may provide insights into the antagonistic function of TLR4 in DCs in allergic inflammation.

Allergen immunotherapy is a therapeutic approach used to induce immune tolerance in patients with allergic diseases. It involves the mediation of multiple molecular, cellular, and humoral pathways that limit allergen-induced early and late-phase inflammatory responses, thereby inducing immunological tolerance. When paired with an allergy vaccination, the ideal adjuvant should promote the stimulation of the innate immune system to enhance the adaptive immunological response to the antigen. Allergen immunotherapy is the only disease-modifying treatment that induces specific immunological memory, thereby providing long-term immunity to the allergen. Physcion, an anthraquinone derivative, induces DC maturation via TLR4 and promotes the differentiation of Th1 cells without affecting the differentiation of Th2 cells, implying that physcion may be useful for treating asthma with Th1/Th2 cell imbalance.^136^ Given the immunosuppressive effects of LPS, monophosphoryl lipid A, a significantly less toxic derivative, is used as an adjuvant in allergen immunotherapy. DC-targeting is a highly effective approach to increasing the immunogenicity of antigens, thereby improving the efficacy of allergen immunotherapy. Amylase-trypsin inhibitors found in wheat may be significant dietary allergen activators and adjuvants due to the marked exacerbation of allergen-specific T-cell proliferation and cytokine production with TLR4 engagement.^137^ Furthermore, nitrated amylase-trypsin inhibitors produced by tetranitromethane cause far greater proliferation of Th cells and production of Th1 and Th2 cytokines than unmodified ones.^138^

It is worth noting that LPS, as a major component of Gram-negative bacteria, is also commonly used in non-infectious lung disease models, such as asthma which is discussed in this section. While LPS can activate the TLR4 signaling pathway in both infectious and non-infectious diseases, the sources of LPS differ, and the resulting immune outcomes are not uniform. In infectious lung diseases, bacteria typically release LPS, which activates the TLR4 signaling pathway and prompts immune cells to eliminate the bacteria. On the other hand, LPS involved in non-infectious lung diseases usually stems from dust and microbial metabolites in the air, present in lower concentrations.^139^ Prolonged exposure to LPS can induce chronic inflammation in the body, which can lead to diseases including asthma, chronic obstructive pulmonary disease,^140^ and pulmonary fibrosis.^141^

### Acute lung injury (ALI)

ALI is a severe respiratory disorder that presents with multiple etiologies characterized by tissue inflammation, pulmonary edema, reduced lung compliance, and widespread capillary leakage. The pathophysiological features of ALI in humans are commonly triggered by excessive inflammatory mediators, of which LPS, a regular pathological factor, is widely used to induce an ALI model.^142^ The various causative factors of ALI include influenza A^143^, hyperoxia,^144^ toxic gas exposure,^145^ ischemia-reperfusion,^146^ acid aspiration,^147^ smoke inhalation,^148^ and high tidal mechanical ventilation.^149^

The positive pro-inflammatory role of activated TLR4 signaling in ALI pathogenesis is well established. The intracellular domain mutation of TLR4 (Tlr4Lps-d), which inhibits TLR4 signaling, showed resistance to LPS challenge.^51^ Genetic silencing of TLR4 considerably reduced ALI, measured by enhanced lung elasticity, decreased edema development, and ameliorated histological alterations.^150^ Collectively, TLR4 has been recognized as an ALI susceptibility gene. It was previously mentioned that different adaptors transmit TLR4 stimulation to cellular responses, such as the MyD88 and TRIF adaptors.^151^ The MyD88-dependent LPS/TLR4 signal pathway activates NF-κB which in turn, causes the up-regulation of pro-inflammatory cytokines such as IL-1β, IL-6, and TNF-α.^152^ TRIF signals either through IKK-ϵ, leading to IRF3 activation, or through TRAF6-mediated NF-κB activation.^153^ In acid-triggered ALI, TLR4-TRIF-TRAF6-NF-κB signaling is a critical pathway connecting acid damage to ALI severity.^150^^,^^154^ Other than LPS, oxidative stress was also implicated in ALI. Possibly due to the unique exposure of the air-liquid interface to an aerobic environment, making it more susceptible to oxidation. This leads to the production of reactive oxygen species and oxidized phospholipids, which are involved in the pathogenesis of ALI.^150^ Furthermore, LPS activation of TLR4 receptor has been shown to induce NADPH oxidase-mediated reactive oxygen species production, resulting in the activation of proinflammatory NF-κB^155^ and TNF-α signaling,^156^^,^^157^ so LPS and oxidative stress are not isolated pathogenesis. During hemorrhagic shock and sepsis-induced ALI, extracellular cold-inducible RNA-binding protein triggers an inflammatory response in DCs by acting as a DAMP. SIIN-CIRP is a fusion protein prepared by combining the amino acids of CD8 T cell's TCR-specific epitope from ovalbumin along with three flanking amino acids with the N-terminal of murine CIRP. This protein induces cytokine production and maturation and migration of DCs through a TLR4-MD2 mediated NF-κB pathway.^158^ LLDT-8, a triptolide derivative, inhibited the activation of DCs in both LPS and *P. aeruginosa*-induced ALI mouse models. Moreover, LLDT-8 influenced the maturation, apoptosis, and cytokine secretion capacity of BMDCs, potentially through modulation of TLR4 expression and NF-κB signaling *in vitro*.^159^

FP7, a TLR4 antagonist, demonstrated protective effects against influenza virus-induced ALI by reducing proinflammatory cytokines (IL-6, IL-8, and MIP-1β) produced by monocytes and DCs. Moreover, FP7 prevented DC maturation by blocking TLR4 stimulation and antagonized TLR4-induced glycolytic activity in human DCs.^107^ Endoplasmic reticulum (ER) stress is characterized by the accumulation of unfolded or misfolded proteins in the endoplasmic reticulum.^160^ Kim et al have demonstrated that such stress contributes to LPS-induced lung inflammation by activating NF-κB signaling and the production of proinflammatory mediators such as TNF-α, IL-1β, chemokine CXCL1, intercellular adhesion molecule-1 (ICAM-1), and vascular endothelial growth factor (VEGF).^161^ Furthermore, they also found that in LPS-induced ALI, there is a positive feedback loop between IL-17A and endoplasmic reticulum stress, which results in an increase in the stress and NF-κB activation.^162^

### Lung cancer

DCs obtained from human non-small cell lung cancer (NSCLC) patients were classified into three groups based on their level of CD11c expression: CD11c^high^ mDC, CD11c^-^ pDC, and a third intermediate group expressing moderate levels of CD11c. CD11c ^high^ tumor-infiltrating DCs displayed a “semi-mature” phenotype when stimulated by TLR4, and they only partially matured and secreted limited amounts of cytokines, indicating a poor antigen-presenting cell function.^163^

**Figure 2:**
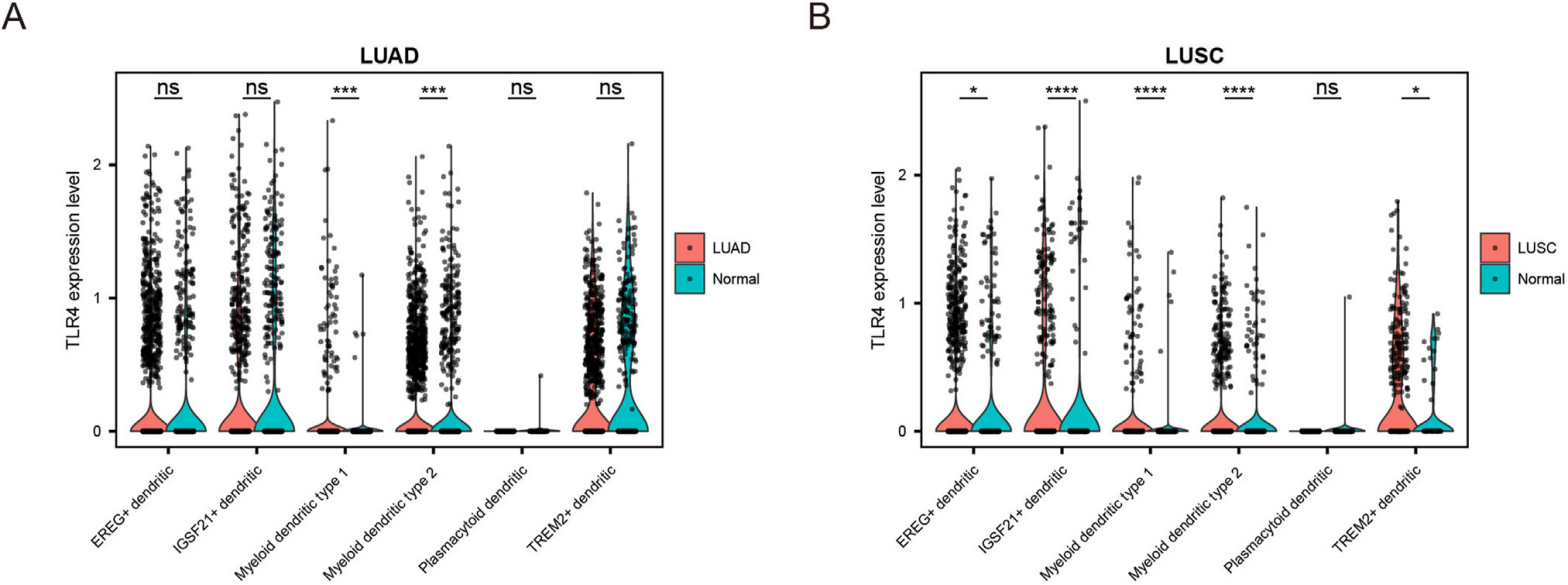
Expression patterns of TLR4 in dendritic cells of normal lung tissues and lung carcinomas. TLR4 expression at the single-cell level in six different dendritic cell subsets stratified by histology in **(A)** lung adenocarcinoma (LUAD) and **(B)** lung squamous cell carcinoma (LUSC) cohorts is visualized in the boxplots. *P*-values were calculated using the Kruskal–Wallis test. ^∗^*P* < 0.05, ^∗∗^*P* < 0.1, ^∗∗∗^*P* < 0.001, ^∗∗∗∗^*P* < 0.0001.

High expression of TLR4 was detected in a majority of lung cancer specimens (mainly NSCLC) compared to tumor-free lung tissue, with TLR4 expression levels positively linked to tumor cell differentiation. This suggests TLR4 may have a dual function in lung cancer, one promoting tumor cell survival, and the other promoting immune defense against malignant transformation.^164^ To further study TLR4 expression on DCs and their subsets, we re-analyzed single-cell RNA sequencing data from primary and adjacent lung adenocarcinoma and lung squamous cell carcinoma samples from Li's cohort^165^ (Fig. 2). All cells were visualized by Uniform Manifold Approximation and Projection. TLR4 expression was elevated in myeloid dendritic type 1 and type 2 in lung adenocarcinoma (Fig. 2A), as well as in EREG^+^DC, IGSF21^+^DC, and myeloid dendritic type 1 and type 2 in lung squamous cell carcinoma tissues (Fig. 2B), compared to normal tissues. The up-regulation of TLR4 expression in DCs may indicate an immune-enhancing effect. Nevertheless, further investigation is required to determine the precise contribution of TLR4 in DCs regarding the pathogenesis and progression of lung tumors.

Various studies have reported that certain regulatory factors mediate the protective function of anti-tumor immunity through TLR4 in DCs. Kaplan–Meier and univariate Cox analyses were conducted for preliminary screening of ferroptosis-related genes with potential prognostic capacity in a cohort analysis on lung adenocarcinoma. According to a risk score analysis, the down-regulation of TLR4 in high-risk groups among the ferroptosis-related 15-gene signature suggests a protective role of TLR4 in lung adenocarcinoma.^166^ In an experimental Lewis lung cancer model, it was found that recombinant human calcineurin B subunit (rhCNB) up-regulated different mature molecules such as CD40, CD80, CD86, and MHCII in DCs, as well as stimulated the formation of CD4^+^ and CD8^+^ T cells in splenocytes from wild-type mice through a TLR4-dependent pathway. Furthermore, the intraperitoneal administration of rhCNB resulted in a 50% reduction in the growth of Lewis lung cancer tumors, and culture supernatants from rhCNB-stimulated immune cells induced apoptosis of Lewis lung cancer cells.^167^ A serum complement lectin named Ficolin-2 was found to be present in considerably lower concentrations in the serum of lung cancer patients than in those of healthy donors. The administration of exogenous Ficolin-2 was observed to effectively inhibit tumor cell growth in murine tumor models by binding to TLR4 in DCs and enhancing their antigen-presenting abilities to CD8^+^ T cells.^168^ The expression of calreticulin on the cell membrane (mCALR) had a favorable link with DC infiltration in NSCLC and had a significant association with the prognosis of NSCLC patients. mCALR promoted the migration and maturation of DCs by activating CALR-TLR4-MyD88 signaling and boosting the production of TNF-α and CCL19. Additionally, it suppressed the progression of lung cancer by promoting the infiltration of DCs within lung cancer tissues *in vivo*.^169^

Despite the potential anti-tumor effects of TLR4 in DCs, several studies have suggested that TLR4 could also mediate tumor-promoting effects. Fridman et al have reviewed the influence of infectious context and immune cell infiltration organization in the progression of human NSCLC. Their study showed that bacterial stimulation of tumor cells through TLR4 might promote tumor survival and induce chemoresistance, which could be protumorigenic.^170^

DCs hold the potential for the stimulation of robust anti-tumor immunity. Despite this potential, the clinical applications of DC-based immunotherapy are restricted by the low potency observed in generating tumor antigen-specific T-cell responses. The utilization of the Mtb heat shock protein X as an immunoadjuvant in DC-based tumor immunotherapy could provide a potential solution for generating potent immunostimulatory DCs. Mtb heat shock protein X stimulates the maturation of DCs, as well as the production of proinflammatory cytokines such as TNF-α, IL-1β, IL-6, and IFN-β, through TLR4 binding which is partially mediated by both the MyD88 and the TRIF signaling pathways. The administration of Mtb heat shock protein X-stimulated DCs improved the activation of naive T cells and promoted tumor-targeted Th1 type and cytotoxic T cell immunity.^171^ Chorismate mutase (Rv1885c), which is a putative *Mtb* virulence factor, also holds promising potential as an immunoadjuvant in DC-based tumor immunotherapy. It functionally activated DCs by up-regulating costimulatory molecules, increasing the production of proinflammatory cytokines, enhancing migration, and triggering the Th1-type immune response dose-dependently through TLR4-mediated signaling. Moreover, the subcutaneous injection of chorismate mutase-activated DCs loaded with cell lysates led to decreased tumor mass, improved mouse survival, and lower tumor incidence in Lewis lung cancer cell-bearing mice. These effects were primarily due to functional cytotoxic T lymphocyte-mediated oncolytic activity which inhibited the proliferation and metastasis-related genes of cancer. Additionally, chorismate mutase-induced DCs have the possibility to produce memory CD4^+^ T cells which demonstrate long-term tumor prevention benefits.^172^

In addition to DC-based immunotherapy, tumor-associated antigen-based vaccines have been developed as potential cancer treatments. However, tumor-associated antigens may have limited therapeutic effectiveness due to their lack of immunogenicity and immune evasion mechanisms present in advanced malignancies. A new vaccine adjuvant system combining the T-cell costimulatory molecule SA-4-1BBL with the TLR4 agonist monophosphoryl lipid was evaluated for its ability to enhance the effects of tumor-associated antigens. This approach was found to be therapeutically effective, as evidenced by enhanced DC activation, improved CD8^+^ T cell function, and increased intratumoral ratio of CD8^+^ T effector cells to CD4^+^ FoxP3^+^ Treg cells.^173^

## Conclusions

Recent studies have revealed important insights into the regulation of LPS/TLR4 signaling, as well as other TLR4-related pathways. These observations have significantly improved our understanding of the function of DCs and the specific role of TLR4 in DCs in lung diseases (Table 3). Given that dysregulation of TLR4 signaling in DCs can lead to a range of immune dysfunctions that contribute to various pulmonary diseases, it is essential to explore these underlying mechanisms in greater detail to identify new targets for treatment.

**Table 3:** Functions and mechanisms of TLR4 in DCs in lung diseases.

Lung diseases	Classification	TLR4 functions in DCs	Mechanisms	Reference(s)
Infectious	Gram-positive bacteria	Induce Th1 and Th17 response	MAPKs, NF-κB, and PI3K-Akt pathways	41
	Gram-negative bacteria	Induce Th1 response	IL-12p70 induction by p38 and JNK1/2 signaling	52
		Enhance NK cell recruitment	NK cell cytotoxicity and IFN-γ secretion	48
		Stimulate B cell IgA response	IL-10 production	174
		Activate Treg cells	IL-10 production	56
		Induce CD8^+^ T cell response	Unmentioned	
	Mycobacterium tuberculosis	Induce Th17 response	MAPK and NF-κB signaling	90
		Induce Th1 response	MyD88 and TRIF signaling collaboration	97
	Virus pathogens	Proinflammatory cytokine production	NF-κB and ERK1/2 MAPK pathways	101
		Induce CD8^+^ T cell response	cDCs, CD11b^+^ and CD103^+^ DCs recruitment and costimulatory molecule CD86 up-regulation	108
Noninfectious	Asthma	Induce Th2 response	TLR4/MyD88 pathway	34
		Induce Th17 response	RAGE-NF-κB signaling	115
		Activate Treg cells	TRIF/IRF3/IFN-β-mediated DC GITRL down-regulation	128
		Induce Th1 response	The expression of CD80 and CD86 elevated skewing the Th1/Th2 balance toward Th1	132
	Acute lung injury	Proinflammatory cytokine production	MyD88 and TRIF signals mediated NF-κB and TNF-α signaling	152, 153, 154,156
	Lung cancer	Induce Th1 and CD8^+^ T cell response	MyD88 and the TRIF signaling pathways	171
		Promote tumor survival and chemoresistance	Unmentioned	

## Author contributions

Writing and original draft preparation: S Xuan, Y Ma, and S Gu. Review and editing: X Zeng. Data curation: H Zhou. Design and funding support: X Zeng, and X Yao. Final approval of manuscript: All authors.

## Conflict of interests

The authors declare that the research was conducted in the absence of any commercial or financial relationships that could be construed as a potential conflict of interest.

## Funding

This research was supported by the 10.13039/501100001809National Natural Science Foundation of China, China (No. 81970016, 81870039).

## References

[1] Murray J.F. (2010). The structure and function of the lung. Int J Tubercul Lung Dis.

[2] Granucci F., Zanoni I. (2009). The dendritic cell life cycle. Cell Cycle.

[3] Baharom F., Thomas S., Rankin G. (2016). Dendritic cells and monocytes with distinct inflammatory responses reside in lung mucosa of healthy humans. J Immunol.

[4] Todate A., Chida K., Suda T. (2000). Increased numbers of dendritic cells in the bronchiolar tissues of diffuse panbronchiolitis. Am J Respir Crit Care Med.

[5] Patel V.I., Metcalf J.P. (2018). Airway macrophage and dendritic cell subsets in the resting human lung. Crit Rev Immunol.

[6] Weibel E.R. (2013). It takes more than cells to make a good lung. Am J Respir Crit Care Med.

[7] Kaisho T. (2012). Pathogen sensors and chemokine receptors in dendritic cell subsets. Vaccine.

[8] Das N.C., Sen Gupta P.S., Biswal S., Patra R., Rana M.K., Mukherjee S. (2022). *In-silico* evidences on filarial cystatin as a putative ligand of human TLR4. J Biomol Struct Dyn.

[9] Arkhypov I., Kurt F.G.Ö., Bitsch R. (2022). HSP90α induces immunosuppressive myeloid cells in melanoma via TLR4 signaling. J Immunother Cancer.

[10] Beutler B., Eidenschenk C., Crozat K. (2007). Genetic analysis of resistance to viral infection. Nat Rev Immunol.

[11] Schreibelt G., Tel J., Sliepen K.H.E.W.J. (2010). Toll-like receptor expression and function in human dendritic cell subsets: implications for dendritic cell-based anti-cancer immunotherapy. Cancer Immunol Immunother.

[12] Gotoh K., Tanaka Y., Nishikimi A. (2010). Selective control of type I IFN induction by the Rac activator DOCK2 during TLR-mediated plasmacytoid dendritic cell activation. J Exp Med.

[13] Desch A.N., Randolph G.J., Murphy K. (2011). CD103^+^ pulmonary dendritic cells preferentially acquire and present apoptotic cell-associated antigen. J Exp Med.

[14] Fossum E., Grødeland G., Terhorst D. (2015). Vaccine molecules targeting Xcr1 on cross-presenting DCs induce protective CD8+ T-cell responses against influenza virus. Eur J Immunol.

[15] del Rio M.L., Rodriguez-Barbosa J.I., Kremmer E., Förster R. (2007). CD103^−^ and CD103^+^ bronchial lymph node dendritic cells are specialized in presenting and cross-presenting innocuous antigen to CD4^+^ and CD8^+^ T cells. J Immunol.

[16] Hintzen G., Ohl L., del Rio M.L. (2006). Induction of tolerance to innocuous inhaled antigen relies on a CCR7-dependent dendritic cell-mediated antigen transport to the bronchial lymph node. J Immunol.

[17] GeurtsvanKessel C.H., Willart M.A., van Rijt L.S. (2008). Clearance of influenza virus from the lung depends on migratory langerin+CD11b- but not plasmacytoid dendritic cells. J Exp Med.

[18] Plantinga M., Guilliams M., Vanheerswynghels M. (2013). Conventional and monocyte-derived CD11b^+^ dendritic cells initiate and maintain T helper 2 cell-mediated immunity to house dust mite allergen. Immunity.

[19] Cao W., Taylor A.K., Biber R.E. (2012). Rapid differentiation of monocytes into type I IFN-producing myeloid dendritic cells as an antiviral strategy against influenza virus infection. J Immunol.

[20] Iijima N., Mattei L.M., Iwasaki A. (2011). Recruited inflammatory monocytes stimulate antiviral Th1 immunity in infected tissue. Proc Natl Acad Sci U S A.

[21] de Heer H.J., Hammad H., Soullié T. (2004). Essential role of lung plasmacytoid dendritic cells in preventing asthmatic reactions to harmless inhaled antigen. J Exp Med.

[22] Kool M., van Nimwegen M., Willart M.A. (2009). An anti-inflammatory role for plasmacytoid dendritic cells in allergic airway inflammation. J Immunol.

[23] Lombardi V., Speak A.O., Kerzerho J., Szely N., Akbari O. (2012). CD8α⁺β⁻ and CD8α⁺β⁺ plasmacytoid dendritic cells induce Foxp3⁺ regulatory T cells and prevent the induction of airway hyper-reactivity. Mucosal Immunol.

[24] Guillerey C., Mouriès J., Polo G. (2012). Pivotal role of plasmacytoid dendritic cells in inflammation and NK-cell responses after TLR9 triggering in mice. Blood.

[25] Krug A., Uppaluri R., Facchetti F. (2002). IFN-producing cells respond to CXCR3 ligands in the presence of CXCL12 and secrete inflammatory chemokines upon activation. J Immunol.

[26] Crozat K., Tamoutounour S., Vu Manh T.P. (2011). Expression of XCR1 defines mouse lymphoid-tissue resident and migratory dendritic cells of the CD8α+ type. J Immunol.

[27] Haniffa M., Shin A., Bigley V. (2012). Human tissues contain CD141^hi^ cross-presenting dendritic cells with functional homology to mouse CD103^+^ nonlymphoid dendritic cells. Immunity.

[28] Peri F., Piazza M., Calabrese V., Damore G., Cighetti R. (2010). Exploring the LPS/TLR4 signal pathway with small molecules. Biochem Soc Trans.

[29] He J., Yuan R., Cui X. (2020). Anemoside B4 protects against *Klebsiella pneumoniae*- and influenza virus FM1-induced pneumonia via the TLR4/Myd88 signaling pathway in mice. Chin Med.

[30] Chen G., Zhang W., Kong L. (2022). Qiguiyin Decoction improves multidrug-resistant *Pseudomonas aeruginosa* infection in rats by regulating inflammatory cytokines and the TLR4/MyD88/NF-*κ*B signaling pathway. BioMed Res Int.

[31] Lin L., Li J., Song Q., Cheng W., Chen P. (2022). The role of HMGB1/RAGE/TLR4 signaling pathways in cigarette smoke-induced inflammation in chronic obstructive pulmonary disease. Immun Inflamm Dis.

[32] Huang M.H., Lin Y.H., Lyu P.C. (2021). Imperatorin interferes with LPS binding to the TLR4 co-receptor and activates the Nrf2 antioxidative pathway in RAW_264.7_ murine macrophage cells. Antioxidants.

[33] Wang M., Xu G., Lü L. (2016). Genetic polymorphisms of IL-17A, IL-17F, TLR4 and miR-146a in association with the risk of pulmonary tuberculosis. Sci Rep.

[34] Hammad H., Plantinga M., Deswarte K. (2010). Inflammatory dendritic cells—not basophils—are necessary and sufficient for induction of Th2 immunity to inhaled house dust mite allergen. J Exp Med.

[35] Tang J., Xu L., Zeng Y., Gong F. (2021). Effect of gut microbiota on LPS-induced acute lung injury by regulating the TLR4/NF-kB signaling pathway. Int Immunopharm.

[36] Li Z., Mao X., Liu Q. (2019). Functional variations of the *TLR4* gene in association with chronic obstructive pulmonary disease and pulmonary tuberculosis. BMC Pulm Med.

[37] Shalaby K.H., Al Heialy S., Tsuchiya K. (2017). The TLR4-TRIF pathway can protect against the development of experimental allergic asthma. Immunology.

[38] Bhattacharyya S., Wang W., Qin W. (2018). TLR4-dependent fibroblast activation drives persistent organ fibrosis in skin and lung. JCI Insight.

[39] Schröder N.W.J., Morath S., Alexander C. (2003). Lipoteichoic acid (LTA) of *Streptococcus pneumoniae*and *Staphylococcus aureus* activates immune cells via Toll-like receptor (TLR)-2, lipopolysaccharide-binding protein (LBP), and CD14, whereas TLR-4 and MD-2 are not involved. J Biol Chem.

[40] Yoshimura A., Lien E., Ingalls R.R., Tuomanen E., Dziarski R., Golenbock D. (1999). Cutting edge: recognition of Gram-positive bacterial cell wall components by the innate immune system occurs via Toll-like receptor 2. J Immunol.

[41] Wu Y., Cui J., Zhang X. (2017). Pneumococcal DnaJ modulates dendritic cell-mediated Th1 and Th17 immune responses through Toll-like receptor 4 signaling pathway. Immunobiology.

[42] Sun X., Wang J., Zhou J. (2017). Subcutaneous immunization with *Streptococcus pneumoniae* GAPDH confers effective protection in mice via TLR2 and TLR4. Mol Immunol.

[43] Roquilly A., Broquet A., Jacqueline C. (2013). Toll-like receptor-4 agonist in post-haemorrhage pneumonia: role of dendritic and natural killer cells. Eur Respir J.

[44] Rietschel E.T., Kirikae T., Schade F.U. (1994). Bacterial endotoxin: molecular relationships of structure to activity and function. Faseb J.

[45] Bertani B., Ruiz N. (2018). Function and biogenesis of lipopolysaccharides. EcoSal Plus.

[46] Akashi S., Shimazu R., Ogata H. (2000). Cell surface expression and lipopolysaccharide signaling via the toll-like receptor 4-MD-2 complex on mouse peritoneal macrophages. J Immunol.

[47] Zhang F.X., Kirschning C.J., Mancinelli R. (1999). Bacterial lipopolysaccharide activates nuclear factor-κB through interleukin-1 signaling mediators in cultured human dermal endothelial cells and mononuclear phagocytes. J Biol Chem.

[48] Alexis N.E., Lay J.C., Almond M., Bromberg P.A., Patel D.D., Peden D.B. (2005). Acute LPS inhalation in healthy volunteers induces dendritic cell maturation *in vivo*. J Allergy Clin Immunol.

[49] van Helden S.F., van den Dries K., Oud M.M. (2010). TLR4-mediated podosome loss discriminates gram-negative from gram-positive bacteria in their capacity to induce dendritic cell migration and maturation. J Immunol.

[50] Kim C.H., Jeong Y.J., Lee J. (2013). Essential role of Toll-like receptor 4 in *Acinetobacter baumannii*-induced immune responses in immune cells. Microb Pathog.

[51] Poltorak A., He X., Smirnova I. (1998). Defective LPS signaling in C3H/HeJ and C57BL/10ScCr mice: mutations in Tlr4 gene. Science.

[52] Agrawal S., Agrawal A., Doughty B. (2003). Different Toll-like receptor agonists instruct dendritic cells to induce distinct Th responses via differential modulation of extracellular signal-regulated kinase-mitogen-activated protein kinase and c-Fos. J Immunol.

[53] Van Elssen C.H., Vanderlocht J., Frings P.W. (2010). *Klebsiella pneumoniae*-triggered DC recruit human NK cells in a CCR5-dependent manner leading to increased CCL19-responsiveness and activation of NK cells. Eur J Immunol.

[54] Evrard B., Balestrino D., Dosgilbert A. (2010). Roles of capsule and lipopolysaccharide O antigen in interactions of human monocyte-derived dendritic cells and *Klebsiella pneumoniae*. Infect Immun.

[55] Li X., Zhang Y.K., Yin B., Liang J.B., Jiang F., Wu W.X. (2019). Toll-like receptor 2 (TLR2) and TLR4 mediate the IgA immune response induced by *Mycoplasma hyopneumoniae*. Infect Immun.

[56] Higgins S.C., Lavelle E.C., McCann C. (2003). Toll-like receptor 4-mediated innate IL-10 activates antigen-specific regulatory T cells and confers resistance to *Bordetella pertussis* by inhibiting inflammatory pathology. J Immunol.

[57] Fallarino F., Pallotta M.T., Matino D. (2015). LPS-conditioned dendritic cells confer endotoxin tolerance contingent on tryptophan catabolism. Immunobiology.

[58] Lu Y.C., Yeh W.C., Ohashi P.S. (2008). LPS/TLR4 signal transduction pathway. Cytokine.

[59] Rogero M.M., Calder P.C. (2018). Obesity, inflammation, Toll-like receptor 4 and fatty acids. Nutrients.

[60] Hong Y.P., Yu J., Su Y.R. (2020). High-fat diet aggravates acute pancreatitis via TLR4-mediated necroptosis and inflammation in rats. Oxid Med Cell Longev.

[61] Pastva A.M., Wright J.R., Williams K.L. (2007). Immunomodulatory roles of surfactant proteins A and D: implications in lung disease. Proc Am Thorac Soc.

[62] Awasthi S., Brown K., King C., Awasthi V., Bondugula R. (2011). A toll-like receptor-4-interacting surfactant protein-A-derived peptide suppresses tumor necrosis factor-α release from mouse JAWS II dendritic cells. J Pharmacol Exp Therapeut.

[63] Ramani V., Madhusoodhanan R., Kosanke S., Awasthi S. (2013). A TLR4-interacting SPA4 peptide inhibits LPS-induced lung inflammation. Innate Immun.

[64] Awasthi S., Singh B., Ramani V., Xie J., Kosanke S. (2019). TLR4-interacting SPA4 peptide improves host defense and alleviates tissue injury in a mouse model of *Pseudomonas aeruginosa* lung infection. PLoS One.

[65] Yu B., Wright S.D. (1996). Catalytic properties of lipopolysaccharide (LPS) binding protein. Transfer of LPS to soluble CD14. J Biol Chem.

[66] Iovine N., Eastvold J., Elsbach P., Weiss J.P., Gioannini T.L. (2002). The carboxyl-terminal domain of closely related endotoxin-binding proteins determines the target of protein-lipopolysaccharide complexes. J Biol Chem.

[67] Gioannini T.L., Teghanemt A., Zhang D., Levis E.N., Weiss J.P. (2005). Monomeric endotoxin: protein complexes are essential for TLR4-dependent cell activation. J Endotoxin Res.

[68] Varshney P., Yadav V., Saini N. (2016). Lipid rafts in immune signalling: current progress and future perspective. Immunology.

[69] Iijima J., Kobayashi S., Kitazume S. (2017). Core fucose is critical for CD14-dependent Toll-like receptor 4 signaling. Glycobiology.

[70] Zanoni I., Ostuni R., Marek L.R. (2011). CD14 controls the LPS-induced endocytosis of Toll-like receptor 4. Cell.

[71] Zanoni I., Ostuni R., Capuano G. (2009). CD14 regulates the dendritic cell life cycle after LPS exposure through NFAT activation. Nature.

[72] Zanoni I., Ostuni R., Barresi S. (2012). CD14 and NFAT mediate lipopolysaccharide-induced skin edema formation in mice. J Clin Invest.

[73] Aksoy E., Taboubi S., Torres D. (2012). The p110δ isoform of the kinase PI(3)K controls the subcellular compartmentalization of TLR4 signaling and protects from endotoxic shock. Nat Immunol.

[74] Wenink M.H., Santegoets K.C., Roelofs M.F. (2009). The inhibitory Fc gamma IIb receptor dampens TLR4-mediated immune responses and is selectively up-regulated on dendritic cells from rheumatoid arthritis patients with quiescent disease. J Immunol.

[75] Ohashi K., Burkart V., Flohé S., Kolb H. (2000). Heat shock protein 60 is a putative endogenous ligand of the toll-like receptor-4 complex. J Immunol.

[76] Smiley S.T., King J.A., Hancock W.W. (2001). Fibrinogen stimulates macrophage chemokine secretion through Toll-like receptor 4. J Immunol.

[77] Paterson H.M., Murphy T.J., Purcell E.J. (2003). Injury primes the innate immune system for enhanced Toll-like receptor reactivity. J Immunol.

[78] Surendran N., Sriranganathan N., Boyle S.M. (2013). Protection to respiratory challenge of *Brucella abortus* strain 2308 in the lung. Vaccine.

[79] World Health Organization. Global Tuberculosis Report 2021. Accessed October 12, 2022. Available at https://www.who.int/publications-detail-redirect/9789240037021.

[80] Rodrigues T.S., Conti B.J., Fraga-Silva T.F.C., Almeida F., Bonato V.L.D. (2020). Interplay between alveolar epithelial and dendritic cells and *Mycobacterium* tuberculosis. J Leukoc Biol.

[81] Wong K.W. (2017). The role of ESX-1 in *Mycobacterium tuberculosis* pathogenesis. Microbiol Spectr.

[82] Gröschel M.I., Sayes F., Simeone R., Majlessi L., Brosch R. (2016). ESX secretion systems: mycobacterial evolution to counter host immunity. Nat Rev Microbiol.

[83] Jang A.R., Kim G., Hong J.J., Kang S.M., Shin S.J., Park J.H. (2019). *Mycobacterium tuberculosis* ESAT6 drives the activation and maturation of bone marrow-derived dendritic cells via TLR4-mediated signaling. Immune Netw.

[84] Sow F.B., Nandakumar S., Velu V. (2011). *Mycobacterium tuberculosis* components stimulate production of the antimicrobial peptide hepcidin. Tuberculosis.

[85] Mazurek J., Ignatowicz L., Kallenius G., Svenson S.B., Pawlowski A., Hamasur B. (2012). Divergent effects of mycobacterial cell wall glycolipids on maturation and function of human monocyte-derived dendritic cells. PLoS One.

[86] Kim W.S., Jung I.D., Kim J.S. (2018). *Mycobacterium tuberculosis* GrpE, a heat-shock stress responsive chaperone, promotes Th1-biased T cell immune response via TLR4-mediated activation of dendritic cells. Front Cell Infect Microbiol.

[87] Kim J.S., Kim W.S., Choi H.G. (2013). *Mycobacterium tuberculosis* RpfB drives Th1-type T cell immunity via a TLR4-dependent activation of dendritic cells. J Leukoc Biol.

[88] Choi S., Choi H.G., Shin K.W. (2018). *Mycobacterium tuberculosis* protein Rv3841 activates dendritic cells and contributes to a T helper 1 immune response. J Immunol Res.

[89] Choi H.H., Kwon K.W., Han S.J. (2019). PPE39 of the *Mycobacterium tuberculosis* strain Beijing/K induces Th1-cell polarization through dendritic cell maturation. J Cell Sci.

[90] Choi H.G., Kim W.S., Back Y.W. (2015). *Mycobacterium tuberculosis* RpfE promotes simultaneous Th1- and Th17-type T-cell immunity via TLR4-dependent maturation of dendritic cells. Eur J Immunol.

[91] Liu Y., Wang R., Jiang J. (2018). A subset of CD1c^+^ dendritic cells is increased in patients with tuberculosis and promotes Th17 cell polarization. Tuberculosis.

[92] Khan N., Vidyarthi A., Pahari S. (2016). Signaling through NOD-2 and TLR-4 bolsters the T cell priming capability of dendritic cells by inducing autophagy. Sci Rep.

[93] Aqdas M., Maurya S.K., Pahari S. (2021). Immunotherapeutic role of NOD-2 and TLR-4 signaling as an adjunct to antituberculosis chemotherapy. ACS Infect Dis.

[94] Fremond C.M., Yeremeev V., Nicolle D.M., Jacobs M., Quesniaux V.F., Ryffel B. (2004). Fatal *Mycobacterium tuberculosis* infection despite adaptive immune response in the absence of MyD88. J Clin Invest.

[95] Fremond C.M., Togbe D., Doz E. (2007). IL-1 receptor-mediated signal is an essential component of MyD88-dependent innate response to *Mycobacterium tuberculosis* infection. J Immunol.

[96] Uehori J., Fukase K., Akazawa T. (2005). Dendritic cell maturation induced by muramyl dipeptide (MDP) derivatives: monoacylated MDP confers TLR2/TLR4 activation. J Immunol.

[97] Orr M.T., Duthie M.S., Windish H.P. (2013). MyD88 and TRIF synergistic interaction is required for TH1-cell polarization with a synthetic TLR4 agonist adjuvant. Eur J Immunol.

[98] Lin S.J., Kuo M.L., Hsiao H.S., Lee P.T. (2016). Azithromycin modulates immune response of human monocyte-derived dendritic cells and CD4^+^ T cells. Int Immunopharm.

[99] Witte A., Wanner G., Sulzner M., Lubitz W. (1992). Dynamics of PhiX174 protein E-mediated lysis of *Escherichia coli*. Arch Microbiol.

[100] Lim J., Koh V.H.Q., Cho S.S.L. (2019). Harnessing the immunomodulatory properties of bacterial ghosts to boost the anti-mycobacterial protective immunity. Front Immunol.

[101] Martinez O., Valmas C., Basler C.F. (2007). Ebola virus-like particle-induced activation of NF-κB and Erk signaling in human dendritic cells requires the glycoprotein mucin domain. Virology.

[102] Okumura A., Pitha P.M., Yoshimura A., Harty R.N. (2010). Interaction between Ebola virus glycoprotein and host toll-like receptor 4 leads to induction of proinflammatory cytokines and SOCS_1_. J Virol.

[103] Escudero-Pérez B., Volchkova V.A., Dolnik O., Lawrence P., Volchkov V.E. (2014). Shed GP of Ebola virus triggers immune activation and increased vascular permeability. PLoS Pathog.

[104] Velayutham T.S., Kolli D., Ivanciuc T., Garofalo R.P., Casola A. (2013). Critical role of TLR4 in human metapneumovirus mediated innate immune responses and disease pathogenesis. PLoS One.

[105] Kolli D., Bao X., Liu T. (2011). Human metapneumovirus glycoprotein G inhibits TLR4-dependent signaling in monocyte-derived dendritic cells. J Immunol.

[106] Shirey K.A., Lai W., Scott A.J. (2013). The TLR4 antagonist Eritoran protects mice from lethal influenza infection. Nature.

[107] Perrin-Cocon L., Aublin-Gex A., Sestito S.E. (2017). TLR4 antagonist FP7 inhibits LPS-induced cytokine production and glycolytic reprogramming in dendritic cells, and protects mice from lethal influenza infection. Sci Rep.

[108] Malloy A.M., Ruckwardt T.J., Morabito K.M., Lau-Kilby A.W., Graham B.S. (2017). Pulmonary dendritic cell subsets shape the respiratory syncytial virus-specific CD8^+^ T cell immunodominance hierarchy in neonates. J Immunol.

[109] Sastry M., Zhang B., Chen M. (2017). Adjuvants and the vaccine response to the DS-Cav1-stabilized fusion glycoprotein of respiratory syncytial virus. PLoS One.

[110] Stoppelenburg A.J., de Roock S., Hennus M.P., Bont L., Boes M. (2014). Elevated Th17 response in infants undergoing respiratory viral infection. Am J Pathol.

[111] Lun S.W.M., Wong C.K., Ko F.W.S., Hui D.S.C., Lam C.W.K. (2009). Expression and functional analysis of toll-like receptors of peripheral blood cells in asthmatic patients: implication for immunopathological mechanism in asthma. J Clin Immunol.

[112] Saito H., Tsurikisawa N., Oshikata C., Tsuburai T., Akiyama K. (2013). Increased interleukin-27 production by antigen-presenting cells promotes regulatory T cell differentiation and contributes to inducing a remission in patients with eosinophilic granulomatosis with polyangiitis. Int Arch Allergy Immunol.

[113] Cho M., Lee J.E., Lim H. (2018). Fibrinogen cleavage products and Toll-like receptor 4 promote the generation of programmed cell death 1 ligand 2-positive dendritic cells in allergic asthma. J Allergy Clin Immunol.

[114] Tjota M.Y., Williams J.W., Lu T. (2013). IL-33-dependent induction of allergic lung inflammation by FcγRIII signaling. J Clin Invest.

[115] Li R., Wang J., Zhu F. (2018). HMGB1 regulates T helper 2 and T helper17 cell differentiation both directly and indirectly in asthmatic mice. Mol Immunol.

[116] Choi J.P., Jeon S.G., Kim Y.K., Cho Y.S. (2019). Role of house dust mite-derived extracellular vesicles in a murine model of airway inflammation. Clin Exp Allergy.

[117] Ishii T., Murakami Y., Narita T. (2022). Myeloid differentiation protein-2 has a protective role in house dust mite-mediated asthmatic characteristics with the proinflammatory regulation of airway epithelial cells and dendritic cells. Clin Exp Allergy.

[118] Derewenda U., Li J., Derewenda Z. (2002). The crystal structure of a major dust mite allergen Der p 2, and its biological implications. J Mol Biol.

[119] Ohto U., Fukase K., Miyake K., Satow Y. (2007). Crystal structures of human MD-2 and its complex with antiendotoxic lipid IVa. Science.

[120] Kim H.M., Park B.S., Kim J.I. (2007). Crystal structure of the TLR4-MD-2 complex with bound endotoxin antagonist eritoran. Cell.

[121] Trompette A., Divanovic S., Visintin A. (2009). Allergenicity resulting from functional mimicry of a Toll-like receptor complex protein. Nature.

[122] Kennedy M.N., Mullen G.E., Leifer C.A. (2004). A complex of soluble MD-2 and lipopolysaccharide serves as an activating ligand for Toll-like receptor 4. J Biol Chem.

[123] Pascoe C.D., Jha A., Basu S. (2020). The importance of reporting house dust mite endotoxin abundance: impact on the lung transcriptome. Am J Physiol Lung Cell Mol Physiol.

[124] Daan de Boer J., JJTH Roelofs, de Vos A.F. (2013). Lipopolysaccharide inhibits Th2 lung inflammation induced by house dust mite allergens in mice. Am J Respir Cell Mol Biol.

[125] Bachus H., Kaur K., Papillion A.M. (2019). Impaired tumor-necrosis-factor-α-driven dendritic cell activation limits lipopolysaccharide-induced protection from allergic inflammation in infants. Immunity.

[126] Kaur K., Bachus H., Lewis C. (2021). GM-CSF production by non-classical monocytes controls antagonistic LPS-driven functions in allergic inflammation. Cell Rep.

[127] Eisenbarth S.C., Piggott D.A., Huleatt J.W., Visintin I., Herrick C.A., Bottomly K. (2002). Lipopolysaccharide-enhanced, Toll-like receptor 4-dependent T helper cell type 2 responses to inhaled antigen. J Exp Med.

[128] Ding F., Liu B., Niu C. (2020). Low-dose LPS induces tolerogenic Treg skewing in asthma. Front Immunol.

[129] Herrick C.A., Bottomly K. (2003). To respond or not to respond: T cells in allergic asthma. Nat Rev Immunol.

[130] Arora M., Poe S.L., Ray A., Ray P. (2011). LPS-induced CD11b^+^Gr1^int^F4/80^+^ regulatory myeloid cells suppress allergen-induced airway inflammation. Int Immunopharm.

[131] Revets H., Pynaert G., Grooten J., De Baetselier P. (2005). Lipoprotein I, a TLR2/4 ligand modulates Th2-driven allergic immune responses. J Immunol.

[132] Haapakoski R., Karisola P., Fyhrquist N. (2013). Toll-like receptor activation during cutaneous allergen sensitization blocks development of asthma through IFN-gamma-dependent mechanisms. J Invest Dermatol.

[133] Nembrini C., Sichelstiel A., Kisielow J., Kurrer M., Kopf M., Marsland B.J. (2011). Bacterial-induced protection against allergic inflammation through a multicomponent immunoregulatory mechanism. Thorax.

[134] Bortolatto J., Borducchi E., Rodriguez D. (2008). Toll-like receptor 4 agonists adsorbed to aluminium hydroxide adjuvant attenuate ovalbumin-specific allergic airway disease: role of MyD88 adaptor molecule and interleukin-12/interferon-gamma axis. Clin Exp Allergy.

[135] Rodríguez D., Keller A.C., Faquim-Mauro E.L. (2003). Bacterial lipopolysaccharide signaling through Toll-like receptor 4 suppresses asthma-like responses via nitric oxide synthase 2 activity. J Immunol.

[136] Hwang Y.H., Kim S.J., Yee S.T. (2020). Physcion-matured dendritic cells induce the differentiation of Th1 cells. Int J Mol Sci.

[137] Bellinghausen I., Weigmann B., Zevallos V. (2019). Wheat amylase-trypsin inhibitors exacerbate intestinal and airway allergic immune responses in humanized mice. J Allergy Clin Immunol.

[138] Ziegler K., Neumann J., Liu F. (2018). Nitration of wheat amylase trypsin inhibitors increases their innate and adaptive immunostimulatory potential *in vitro*. Front Immunol.

[139] Tang B., Christia C., Malarvannan G. (2020). Legacy and emerging organophosphorus flame retardants and plasticizers in indoor microenvironments from Guangzhou, South China. Environ Int.

[140] Kim H.Y., Lee H.S., Kim I.H. (2022). Comprehensive targeted metabolomic study in the lung, plasma, and urine of PPE/LPS-induced COPD mice model. Int J Mol Sci.

[141] Yang H., Hua C., Yang X. (2020). Pterostilbene prevents LPS-induced early pulmonary fibrosis by suppressing oxidative stress, inflammation and apoptosis *in vivo*. Food Funct.

[142] Lee W.L., Downey G.P. (2001). Neutrophil activation and acute lung injury. Curr Opin Crit Care.

[143] Narasaraju T., Yang E., Samy R.P. (2011). Excessive neutrophils and neutrophil extracellular traps contribute to acute lung injury of influenza pneumonitis. Am J Pathol.

[144] Dias-Freitas F., Metelo-Coimbra C., Roncon-Albuquerque R. (2016). Molecular mechanisms underlying hyperoxia acute lung injury. Respir Med.

[145] Shohrati M., Karimzadeh I., Saburi A., Khalili H., Ghanei M. (2014). The role of N-acetylcysteine in the management of acute and chronic pulmonary complications of sulfur mustard: a literature review. Inhal Toxicol.

[146] Zhao W., Zhou S., Yao W. (2014). Propofol prevents lung injury after intestinal ischemia-reperfusion by inhibiting the interaction between mast cell activation and oxidative stress. Life Sci.

[147] Mizushina Y., Karasawa T., Aizawa K. (2019). Inflammasome-independent and atypical processing of IL-1β contributes to acid aspiration-induced acute lung injury. J Immunol.

[148] Enkhbaatar P., Traber D. (2004). Pathophysiology of acute lung injury in combined burn and smoke inhalation injury. Clin Sci.

[149] Brochard L., Slutsky A., Pesenti A. (2017). Mechanical ventilation to minimize progression of lung injury in acute respiratory failure. Am J Respir Crit Care Med.

[150] Imai Y., Kuba K., Neely G.G. (2008). Identification of oxidative stress and Toll-like receptor 4 signaling as a key pathway of acute lung injury. Cell.

[151] Akira S., Uematsu S., Takeuchi O. (2006). Pathogen recognition and innate immunity. Cell.

[152] Wang N., Geng C., Sun H., Wang X., Li F., Liu X. (2019). Hesperetin ameliorates lipopolysaccharide-induced acute lung injury in mice through regulating the TLR4-MyD88-NF-κB signaling pathway. Arch Pharm Res (Seoul).

[153] Sato S., Sugiyama M., Yamamoto M. (2003). Toll/IL-1 receptor domain-containing adaptor inducing IFN-β (TRIF) associates with TNF receptor-associated factor 6 and TANK-binding kinase 1, and activates two distinct transcription factors, NF-κB and IFN-regulatory factor-3, in the Toll-like receptor signaling. J Immunol.

[154] Vermeulen L., De Wilde G., Notebaert S., Vanden Berghe W., Haegeman G. (2002). Regulation of the transcriptional activity of the nuclear factor-κB p65 subunit. Biochem Pharmacol.

[155] Park H.S., Jung H.Y., Park E.Y., Kim J., Lee W.J., Bae Y.S. (2004). Direct interaction of TLR4 with NAD(P)H oxidase 4 isozyme is essential for lipopolysaccharide-induced production of reactive oxygen species and activation of NF-κB. J Immunol.

[156] Matsubara T., Ziff M. (1986). Increased superoxide anion release from human endothelial cells in response to cytokines. J Immunol.

[157] Murphy H.S., Shayman J.A., Till G.O. (1992). Superoxide responses of endothelial cells to C5a and TNF-alpha: divergent signal transduction pathways. Am J Physiol.

[158] Aziz M., Brenner M., Wang P. (2019). Extracellular CIRP (eCIRP) and inflammation. J Leukoc Biol.

[159] Chen Y., Kuang Z., Wei W. (2022). Protective role of (5R)-5-hydroxytriptolide in lipopolysaccharide-induced acute lung injury by suppressing dendritic cell activation. Int Immunopharm.

[160] Hosoi T., Ozawa K. (2009). Endoplasmic reticulum stress in disease: mechanisms and therapeutic opportunities. Clin Sci.

[161] Kim H.J., Jeong J.S., Kim S.R., Park S.Y., Chae H.J., Lee Y.C. (2013). Inhibition of endoplasmic reticulum stress alleviates lipopolysaccharide-induced lung inflammation through modulation of NF-κB/HIF-1α signaling pathway. Sci Rep.

[162] Kim S.R., Kim H.J., Kim D.I. (2015). Blockade of interplay between IL-17A and endoplasmic reticulum stress attenuates LPS-induced lung injury. Theranostics.

[163] Perrot I., Blanchard D., Freymond N. (2007). Dendritic cells infiltrating human non-small cell lung cancer are blocked at immature stage. J Immunol.

[164] Zhang Y.B., He F.L., Fang M. (2009). Increased expression of Toll-like receptors 4 and 9 in human lung cancer. Mol Biol Rep.

[165] Zhang L., Zhang Y., Wang C. (2022). Integrated single-cell RNA sequencing analysis reveals distinct cellular and transcriptional modules associated with survival in lung cancer. Signal Transduct Targeted Ther.

[166] Zhang A., Yang J., Ma C., Li F., Luo H. (2021). Development and validation of a robust ferroptosis-related prognostic signature in lung adenocarcinoma. Front Cell Dev Biol.

[167] Yang J., Zhang H., Zhu Z., Gao Y., Xiang B., Wei Q. (2019). The immunostimulatory effects and pro-apoptotic activity of rhCNB against Lewis lung cancer is mediated by Toll-like receptor 4. Cancer Med.

[168] Ding Q., Shen Y., Li D. (2017). Ficolin-2 triggers antitumor effect by activating macrophages and CD8^+^ T cells. Clin Immunol.

[169] Chen R., Huang M., Yang X. (2021). CALR-TLR4 complex inhibits non-small cell lung cancer progression by regulating the migration and maturation of dendritic cells. Front Oncol.

[170] Sautès-Fridman C., Cherfils-Vicini J., Damotte D. (2011). Tumor microenvironment is multifaceted. Cancer Metastasis Rev.

[171] Jung I.D., Shin S.J., Lee M.G. (2014). Enhancement of tumor-specific T cell-mediated immunity in dendritic cell-based vaccines by *Mycobacterium tuberculosis* heat shock protein X. J Immunol.

[172] Jeong H., Lee S.Y., Seo H., Kim D.H., Lee D., Kim B.J. (2022). Potential of *Mycobacterium tuberculosis* chorismate mutase (Rv1885c) as a novel TLR4-mediated adjuvant for dendritic cell-based cancer immunotherapy. OncoImmunology.

[173] Srivastava A.K., Dinc G., Sharma R.K., Yolcu E.S., Zhao H., Shirwan H. (2014). SA-4-1BBL and monophosphoryl lipid A constitute an efficacious combination adjuvant for cancer vaccines. Cancer Res.

[174] Jiang Z., Song F., Li Y. (2017). Capsular polysaccharide is a main component of *Mycoplasma ovipneumoniae* in the pathogen-induced toll-like receptor-mediated inflammatory responses in sheep airway epithelial cells. Mediat Inflamm.

